# Social Determinants of Health and Well-Being of Adolescents in Multicultural Families in South Korea: Social-Cultural and Community Influence

**DOI:** 10.3389/fpubh.2021.641140

**Published:** 2021-03-24

**Authors:** Jungeun Shin, Hyeonkyeong Lee, Eun Kyoung Choi, Chungmo Nam, Sun-Mi Chae, Oksik Park

**Affiliations:** ^1^Department of Nursing, Yeoju Institute of Technology, Gyeonggi-do, South Korea; ^2^College of Nursing, Moim Kim Nursing Research Institute, Yonsei University, Seoul, South Korea; ^3^Department of Preventive Medicine and Public Health, Yonsei University, Seoul, South Korea; ^4^College of Nursing and the Research Institute of Nursing Science, Seoul National University, Seoul, South Korea; ^5^Korea Association for Supporting Youth from Multicultural Families, Seoul, South Korea

**Keywords:** cultural diversity, adolescents, well-being, social determinants of health, social environment

## Abstract

**Objectives:** Adolescents in multicultural families (AMFs) are exposed to numerous stressors and face environmental vulnerability within the family, school, and community systems, which may affect their health and well-being. Concrete discussion on policies is lacking due to insufficient data on the levels of well-being of AMFs in South Korea. This study aimed to investigate social-cultural and community factors affecting their well-being.

**Methods:** A cross-sectional study was conducted with a convenience sample of 206 AMFs (aged 13–18 years) from 16 general schools and three multicultural schools across eight large cities. AMFs completed a self-administrative questionnaire assessing well-being, individual factors (acculturative stress, health behavior), social and community factors (social support, sense of community), and environmental factors (school type, economic status). Data were analyzed using structural equation modeling.

**Results:** Social support and sense of community significantly and directly affected well-being. The economic status and type of school had an indirect effect on well-being, whereas the effect of acculturative stress was not significant. Factors significantly affecting adolescents' well-being were social support, sense of community, economic status, and type of school.

**Conclusion:** Addressing well-being may be the strategy leading AMFs to grow into healthy adults. These results could help educators, health professionals, and policymakers to identify ways to enhance the well-being of AMFs.

## Introduction

Social determinants of health (SDH) lead to the difference in health outcomes depending on the distribution of capital, power, and resources (1), which are highlighted as the sources of health disparities in populations. SDH are defined as broad social and economic conditions that determine the quality of individual health outcomes (2). Based on the model proposed by Dahlgren and Whitehead (2) and our systematic review of the literature, we classify the SDH affecting the well-being of AMFs into individual, social and community network, and environmental factors.

The population of multicultural families consisting of a marriage immigrant or foreigner with Korean citizenship,have been growing at a rapid pace in South Korea since 2000. Consequently, the number of adolescents in multicultural families (AMFs) was about 122,2000 in 2018 in South Korea and tends to continuously increase annually (3). AMFs accounted for 2.2% of the total Korean adolescent population in 2018, which is three times higher than that in 2012 (0.7%) (3). However, AMFs have demonstrated a lower subjective well-being than the native Korean youth. According to the Youth Health Behavior Web-based Survey in 2013 (4), only 56.3% of AMFs reported subjective happiness, 34.5% experienced depression, and 19.8% had suicidal ideation (5). AMFs also faced daily discrimination, acculturation stress, psychological maladjustment, parent-child conflict, bullying, and the tendency to drop out of school (5, 6).

This study shows the relationship between how AMFs experience the SDH in the contexts of family, school, and community surrounding their daily lives and their well-being. It may be fundamental to address the social determinants of health to close health equity gaps of such a marginalized population in the South Korean society in which the proportion of AMF within the total adolescent population are expected to increase gradually. In an ongoing research improving well-being of AMFs, it is suggested to integrate SDH including factors related to family, school, and community environment.

Assessing the level of well-being of AMFs allows the detection of any early signs of worsening well-being to prevent the potential risk of being marginalized as a vulnerable class (7). As positive effects of adolescents well-being on life outcomes such as decreased problems and increased healthy adjustment to adulthood (8) and also beneficial health outcomes in young adulthood (9) have been recognized, improving the well-being of AMFs is fundamental to close health equity gaps in their future adult life. However, there is a lack of attention on well-being of AMF in South Korea and insufficient empirical evidence for the well-being levels and its determinants.

The SDH among adolescents are strongly affected by individual characteristics, peers, family, community, society, and country (10) and can be explained by social and environmental factors accounting for 75% of population health (11). Unless resolving concerns such as low incomes in vulnerable populations, the negative influences of neighborhoods, and educational inequalities, these SDH can widen the health gap and increase disease burden and medical expenditure (12). Adolescents who are vulnerable are frequently exposed to risky situations during their growth that could affect their physical, psychological, and social well-being throughout their lifespans (13).

Lower well-being and health in AMFs may be linked to individual, cultural, and environmental factors (14). AMFs tend not to receive sufficient family support as their immigrant parents may face problems such as marital conflict, depression, and language-related difficulties (15). Being constantly exposed to potential problems, AMFs face high stress and may have insufficient access to community resources to help overcome stress (16). Immigrant youths are also observed to have low social-economic status (17). However, there is limited research addressing the relationships between family, school, and community systems, environmental health, and the well-being of AMFs.

In summary, well-being can be viewed as the result of mutually complex relationships influenced by individual, family, and environmental factors rather than as problems related to specific diseases (18, 19). A better understanding of SDH factors that affect AMFs' well-being may facilitate the development of programs and policies for improvement.

The individual SDH factors related to the well-being of AMFs refer to psychological conflicts, acculturative stress, low self-esteem, depression, and discrimination in daily life (20–22). Social and community network factors include social support and sense of community (SOC). Social support by peers, family, and teachers is the most important factor affecting well-being among adolescents, and greater social support results in stable school lives and higher self-esteem (23) and consequently increases youth's well-being (24). SOC is known to be a significant SDH and communities can contribute positively to the formation of personal and social identities in adolescents which can have a significant impact on the communities themselves. SOC includes residence-related factors, regional characteristics, as well as community engagement and attitudes (25). The environmental factors of SDH include the factors related to family (economic status of the family) and school (school type).

**Figure 1:**
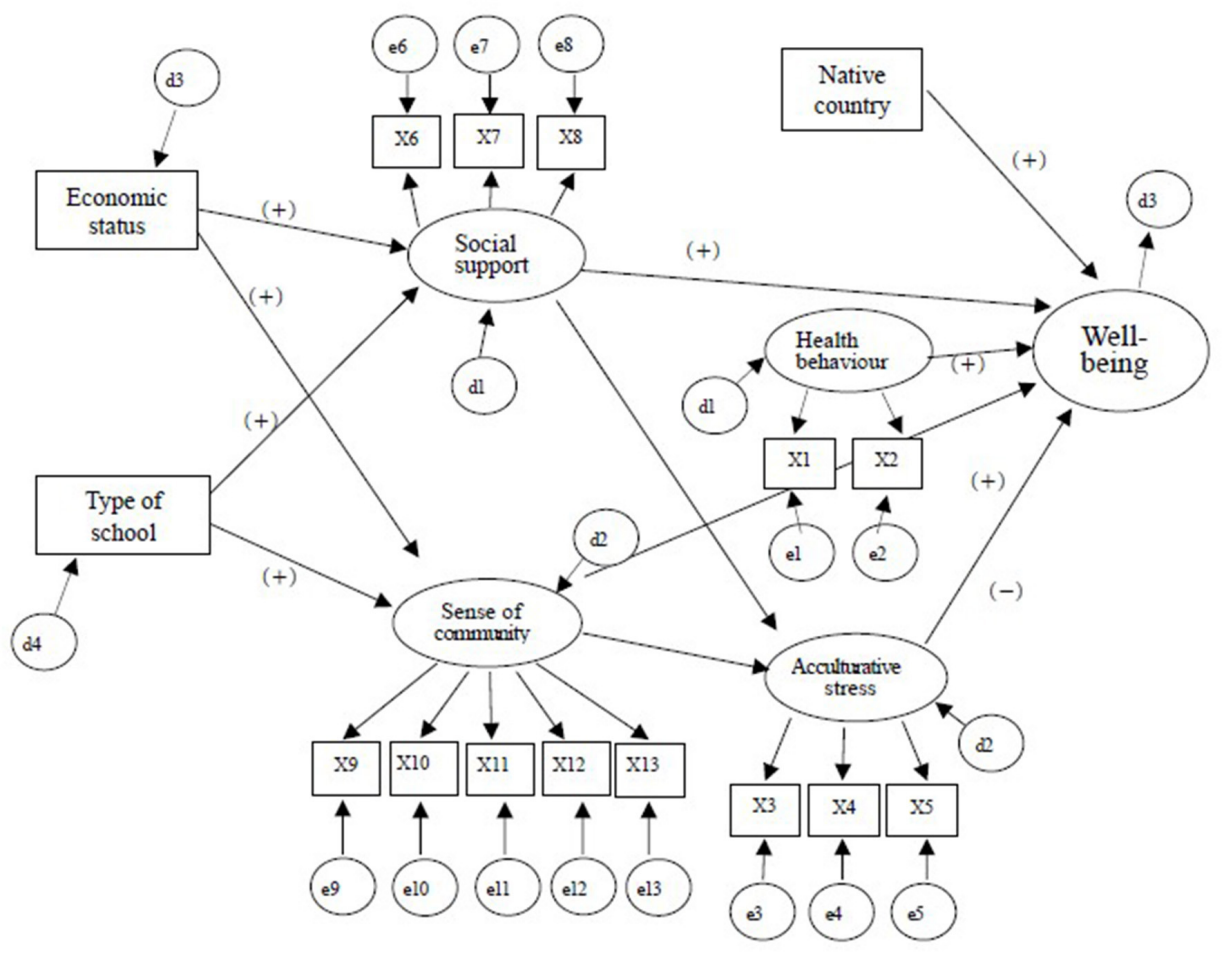
Hypothesized structural equation model. X1: Health behavior and problem, X2: Eating habits, X3: Perceived discrimination, X4: Korean language skill, X5: Feel isolated, X6: Peer support, X7: Family support, X8: Teacher support, X9: Satisfaction of needs and opportunities for involvement, X10: Support and emotional connection with peers, X11: Support and emotional connection in the community. X12: Sense of belonging, X13: Opportunities for influence.

The purpose of the study was to identify how SDH factors such as individual factors (native country, health behavior, acculturative stress), social and community network factors (social support, SOC), and environmental factors (economic status, type of school) affect well-being of AMFs in South Korea. We hypothesized that (i) individual factors would have direct and indirect effects on well-being, (ii) social and community network factors would have direct effects on well-being, and (iii) environmental factors would have direct effects on well-being. The hypothesized model is presented in Figure 1.

## Method

### Participants

Participants were 206 AMFs currently living in South Korea, who were (i) selected across eight cities from 16 regular and three multicultural schools, which are established for children of multicultural families, (ii) aged 13–18 years, and (iii) eligible as per the definition of Korea's *Multicultural Families Support Act* in 2011 (children of families comprising either foreigners who have South Korean citizenship or marriage immigrants). We excluded (i) refugees, (ii) AMFs with severe physical disabilities or mental health issues, and (iii) North Korean defectors as previous studies suggest that they tend to experience post-traumatic stress disorder from the process of entering South Korea (26). Based on Soper's calculation (27), a minimum sample size of 173 was needed with effect size 0.3, power = 0.8, latent variables = 5, observed variables = 16, and probability level = 0.05. We recruited 206 AMFs to potentially dropping out or providing missing or unclear information.

To obtain a representative sample of AMFs living in diverse areas in South Korea, we selected eight provinces using convenience sampling considering the regional distribution rate on the basis of a national multicultural school list registered with the Ministry of Education. The majority of participants were recruited in-person (*n* = 145, 70.4%), followed by postal mail (*n* = 33, 16%), and e-mail (*n* = 28, 13.6%). We included 206 participants in our analysis after excluding 11 who missed at least one response to the questionnaire.

### Measures

A structured questionnaire was translated from Korean to Chinese, Vietnamese, and Russian by experienced professional translators. The instruments originally developed in English were independently translated to Korean by three bilingual experts and the content was reviewed and finalized by five experts including a director of multicultural family support center, a teacher from a multicultural alternative school and three nursing professors with multicultural education and research experiences. Prior to the survey, a pre-test was conducted with 25 AMFs to confirm that the questionnaire was found to be comprehensible and easy to respond.

### Sociodemographic Characteristics

Sociodemographic characteristics included age, grade, gender, height, weight, and country of birth. Duration of residence was measured with the question: “How long have you lived in South Korea?” AMFs self-reported the structured survey.

### Well-Being

In this study, well-being is a subjective and multidimensional concept considered to reflect AMFs' overall physical, psychological, and social status and social functioning in their environment. Well-being was measured using a short version of PERMA-Profiler (28), which was initially developed to measure adolescents' well-being based on five pillars of well-being: PERMA (Positive emotion, Engagement, Relationships, Meaning, and Accomplishment) (18). We translated the instrument from English into Korean and checked the validity through back-translation. The 23-item short version comprises dimensions of positive emotion (three items), engagement (three items), relationships (three items), meaning (three items), accomplishment (three items), negative emotion (three items), health (three items), loneliness (one item), and happiness (one item). Each item was rated on an 11-point scale (0 = Not at all, 10 = Completely). Higher ratings reflect greater levels of well-being. In this study, the scale demonstrated good reliability with Cronbach's α of 0.92.

### Acculturative Stress

Acculturative stress was measured using the modified version of Acculturative Stress Scale for AMFs (29). This instrument was originally developed by Sandhu and Asrabadi (30), and we used the Korean version. The Acculturative Stress Scale for AMFs consists of 17 items comprising three subdomains: perceived discrimination (10 items), Korean skills (four items), and sense of alienation (three items). The items are rated on a 5-point Likert scale (1 = Strongly disagree, 5 = Strongly agree), where a higher mean score indicates higher stress of cultural adaptation. The scale demonstrated good reliability with Cronbach's α of 0.95.

### Health Behavior

Based on items from the Youth Health Behavior Web-based Survey (2), we included dietary and health behaviors such as physical activity, watching TV, using the internet or the cell phone (within the past 7 days), alcohol consumption, and smoking (in the past year). For health behaviors, we assessed the total time respondents spent doing in these behaviors. For dietary behaviors, we included seven items, asking about the frequency of healthy and unhealthy food intake. The responses were rated using on a 5-point Likert scale (0 = never, 5 = routinely). We used factor analysis for all items (Kaiser-Meyer-Olkin = 0.60, *p* < 0.001) and included them in structural equation modeling.

### Social Support

To measure the participants' social support, this study used the Social Support scale developed by Han and Yoo (31). The instrument consists of eight items in three subdomains: family support (eight items), peer support (eight items), and teacher support (eight items). The items are rated on a five-point Likert scale from one (Strongly disagree) to five (Strongly agree), and a higher score reflects greater social support. In this study, Cronbach's α was 0.92.

### Sense of Community

We used the 20-item Brief Scale of Sense of Community in Adolescents (32), which was translated to Korean through a committee translation method. It consists of five subdomains: community participation and satisfaction, support and emotional connection with peers, support and emotional connection with neighbors, sense of belonging, and opportunity for involvement (25). Each item was rated on a 5-point Likert scale (0 = Not at all true, 4 = Completely true) and a higher score indicates a greater SOC with Cronbach's α of 0.95.

### Environmental Factors

Environmental factors were assessed using economic status (based on an annual income of family) and type of school (general school or multicultural alternative school), and included in the SEM as observed variables.

### Data Collection

This study was approved by the Institutional Review Board of Yonsei University Health System (No. Y-2018-0132). Data collection occurred between March and May 2019. The principal investigator visited schools, introduced the research methods, data collection, and contents to teachers in charge of AMFs, and posted recruitment flyers on the bulletin board of schools. Also, we delivered the study objectives, methods, expected outcomes, and confidentiality and anonymity to AMFs, and their parents or legal guardians via the school newsletter. All participants and their parents or legal guardians provided written informed consent prior to the survey. The participants who responded to the survey were provided with a gift card worth about 4 USD.

### Data Analysis

Data were analyzed with SPSS version 23.0 and AMOS version 25.0 (IBM, Armonk, NY, USA). The normality of major variables was tested using means, standard deviation, skewness (<3.0), and kurtosis (<10.0), and multicollinearity among variables was examined with coefficient, tolerance (>0.1), and variance inflation factor (<10.0).

The validity of the SEM was checked through a confirmatory factor analysis (CFA). Criteria for model fit included: chi-square test, normed chi-square test (χ^2^ ≤ 3.0, good fit), goodness of fit index (GFI; value > 0.9 indicates acceptable model fit), root mean square error of approximation (RMSEA; value ≤ 0.08 indicates acceptable model fit), comparative fit index (CFI; value > 0.90, good model fit), and parsimonious goodness of fit index (PGFI; values between 0.6 and 0.9 indicate acceptable fit). Bootstrapping was used to verify the significance of direct, indirect, and total effects of the hypothetical model.

## Results

### Sociodemographic Characteristics

The mean age of AMFs was 15.81 ± 1.71 years; 46.6% were boys (*n* = 99), and 53.4% were girls (*n* = 110). The mean body mass index (BMI) was 20.56 ± 3.29 kg/m^2^ with 50.0% of the sample being underweight (BMI < 18.5), and 41.7% having normal weight (18 < BMI < 25.0). The most common country of birth was South Korea (45.6%), followed by China (20.9%), Vietnam (11.7%), and Uzbekistan (3.9%). The most common country of father's origin was South Korea (66.5%), followed by China (10.7%), while the most common country of mother's origin was China (26.7%), followed by Vietnam (21.8%). The majority of AMFs lived in two-parent families (70.4%). In terms of the economic status, 24.8% multicultural families were in the lowest income range ( ≤ 20 million won/year), followed by 20.4% in the lower-middle income range (20–30 million won/year). Of AMFs, 72.8% attended general schools and 27.2% attended multicultural alternative schools (Table 1).

**Table 1:** Socio-demographics of the participants.

**Characteristic**	**Category**	***n***	**%**
Age (years)	13–15	126	61.2
	16–18	80	38.8
Sex	Boy	99	46.6
	Girl	110	53.4
Body mass index (kg/m^2^)	Low	103	50.0
	Normal	86	41.7
	High	17	8.3
Country of birth	South Korea	94	45.6
	China	43	20.9
	Vietnam	24	11.7
	Uzbekistan	19	9.2
	Others	26	12.8
Country of father's origin	South Korea	137	66.5
	China	22	10.7
	Uzbekistan	10	4.9
	Others	20	9.8
	Unknown	15	7.3
Country of mother's origin	China	55	26.7
	Vietnam	45	21.8
	South Korea	18	8.7
	Uzbekistan	17	8.3
	Japan	14	6.8
	Others	34	16.6
	Unknown	23	11.2
Type of family structure	Traditional	145	70.4
	Single parent	27	13.1
	Grand parent	4	1.9
	Stepfamily	30	14.6
Economic status	≤20	51	24.8
(million won/year)	20–30	42	20.4
	30–40	31	15.0
	40–50	17	8.3
	≥50	27	13.1
	Unknown	38	18.4
Type of school	General	150	72.8
	Multicultural	56	27.2

*N = 206. Participants were on average 15.1 years old (SD = 1.71), and Body Mass Index (kg/m^2^) was on average 20.5 (SD = 3.2)*.

### Identification of SEM Model

**Figure 2:**
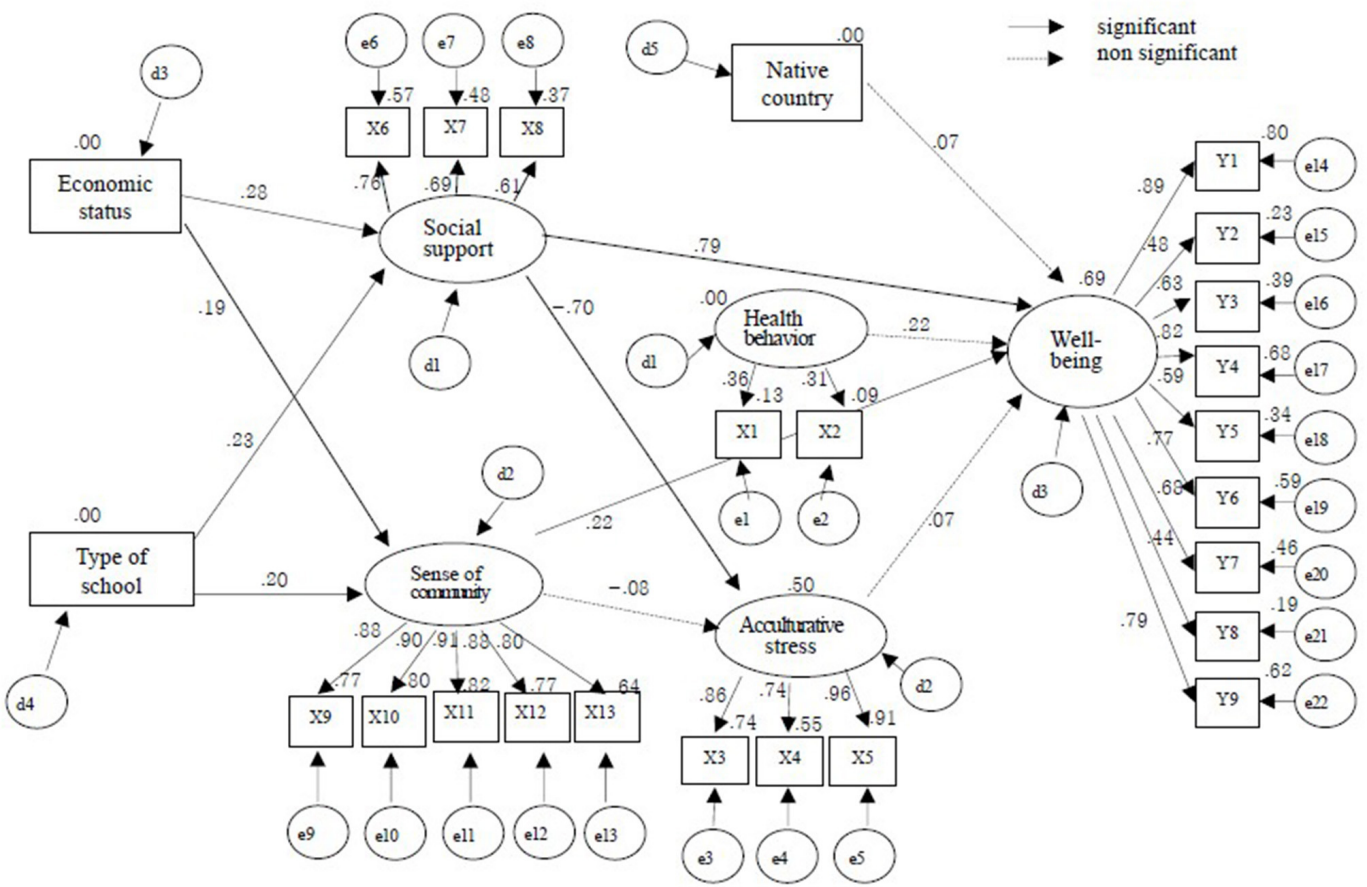
Structural equation model.

In the CFA, the hypothesized measurement model demonstrated an acceptable fit (χ2 = 329.419, *p* < 0.001; Normed χ2 = 2.046; GFI = 0.86; RMSEA = 0.07; CFI = 0.94; PGFI = 0.79). The factor loading of CFA is presented in Table 2. The structural model with all the hypothesized relationships is presented in Figure 2. The model had acceptable fit indices (χ^2^ = 678.578, *p* < 0.001; Normed χ^2^ = 2.570; GFI = 0.78; RMSEA = 0.08; CFI = 0.86; PGFI = 0.76). Bootstrapping was used to analyze the direct effect, indirect effect, total effect, and fit of the model between variables, and is presented in Table 3 and Figure 2.

**Table 2:** Factor loading of confirmatory factor analysis.

**Latent variables**	**Observed variables**	**B**	**β**	**S.E**.	**C.R**.	***p***	**AVE**	**CR**
Acculturative	X1	0.727	0.868	0.041	17.802	<0.001	0.807	0.945
Stress	X2	0.945	0.745	0.069	13.678	<0.001	-	-
	X3	1.000	0.960	-	-	-	-	-
Social support	X4	1.172	0.749	0.136	8.613	<0.001	1.33	1.09
	X5	1.281	0.721	0.153	8.379	<0.001	-	-
	X6	1.000	0.642	-	-	-	-	-
Sense of	X7	1.000	0.909	-	-	-	1.04	1.00
Community	X8	1.054	0.895	0.053	19.811	<0.001	-	-
	X9	1.100	0.880	0.072	15.347	<0.001	-	-
	X10	0.912	0.800	0.059	15.578	<0.001	-	-
	X11	1.032	0.879	0.054	18.979	<0.001	-	-
Well-being	Y1	0.947	0.904	0.061	15.478	<0.001	0.887	0.926
	Y2	0.576	0.505	0.078	7.411	<0.001	-	-
	Y3	0.579	0.651	0.058	9.971	<0.001	-	-
	Y4	0.808	0.843	0.058	14.014	<0.001	-	-
	Y5	0.554	0.611	0.060	9.232	<0.001	-	-
	Y6	0.793	0.789	0.062	12.786	<0.001	-	-
	Y7	0.815	0.702	0.074	10.964	<0.001	-	-
	Y8	0.712	0.464	0.106	6.736	<0.001	-	-
	Y9	1.000	0.806	-	-	-	-	-
Model fit: χ^2^ = 329.419, Normed χ^2^ = 2.046, GFI = 0.860, RMSEA = 0.071, CFI = 0.942, PGFI = 0.757								

*B, Non-standardized regression weight; SE, Standard error; β, Standardized regression weight; AVE, Average Variance Extracted; CR, Critical Ratio*.

*X1, Perceived discrimination; X2, Korean language skill; X3, Feel isolated; X4, Peer support; X5, Family support; X6, Teacher support; X7, Support and emotional connection in the community; X8, Support and emotional connection with peers; X9, Satisfaction of needs and opportunities for involvement; X10, Opportunities for influence X11, Sense of belonging; Y1, Positive emotion; Y2, Negative emotion; Y3, Engagement; Y4, Relationships; Y5, Accomplishment; Y6, Meaning; Y7, Health; Y8, Loneliness; Y9, Happiness*.

**Table 3:** Standardized direct, indirect, and total effects for the hypothetical model.

**Endogenous variables**	**Exogenous variables**	**Direct effect (*p*)**	**Indirect effect (*p*)**	**Total effect (*p*)**
Acculturative	Social support	−0.698 (0.006)	-	−0.698 (0.006)
Stress	Sense of community	−0.075 (0.246)	-	−0.075 (0.246)
	Economic status	-	−0.206 (0.018)	−0.206 (0.018)
	Type of school	-	−0.173 (0.011)	−0.173 (0.011)
Social support	Economic status	0.275 (0.014)	-	0.275 (0.014)
	Type of school	0.226 (0.007)	-	0.226 (0.007)
Sense of community	Economic status	0.190 (0.023)	-	0.190 (0.023)
	Type of school	0.201 (0.007)	-	0.201 (0.007)
Well-being	Native country	0.068 (0.455)	-	0.068 (0.455)
	Health behavior	0.224 (0.185)	-	0.224 (0.185)
	Acculturative stress	0.067 (0.427)	-	0.067 (0.427)
	Social support	0.792 (0.005)	−0.047 (0.457)	0.745 (0.002)
	Sense of community	0.220 (0.019)	−0.005 (0.341)	0.215 (0.019)
	Economic status	-	0.246 (0.010)	0.246 (0.010)
	Type of school	-	0.211 (0.007)	0.211 (0.007)

The environmental factors of AMFs, namely the economic status (β = 0.275, *p* = 0.014) and type of school (β = 0.226, *p* = 0.007), had a direct effect on social support. Social support had a direct effect on acculturative stress (β = −0.698, *p* = 0.006). SOC had a significant direct effect on economic status (β = 0.190, *p* = 0.023) and school type (β = 0.201, *p* = 0.007) in the environmental factors of AMFs. Social support (β = 0.792, *p* = 0.005) and SOC (β = 0.220, *p* = 0.019) also had a significant direct effect on AMFs' well-being. Economic status (β = 0.246, *p* = 0.010) and type of school (β = 0.211, *p* = 0.007) had an indirect effect on well-being, whereas the indirect effect of acculturative stress was not significant. The factors that had a significant effect on AMFs' well-being were social support (β = 0.745, *p* = 0.002), SOC (β = 0.215, *p* = 0.019), economic status (β = 0.246, *p* = 0.010), and type of school (β = 0.211, *p* = 0.007), whereas country of birth, health behavior, and acculturative stress were not significant.

## Discussion

This study investigated the factors that affect the well-being of AMFs based on SDH established by Dalhgren and Whitehead (2). The results of SEM analyses with 206 AMFs support our hypothesized model, confirming that the factors influencing AMFs' well-being were social and community network factors (social support, SOC) and environmental factors (economic status, type of school). In such a country whose society has recently transformed into a multicultural one, there is a lack of empirical evidence on the social and environmental impacts on the health of the multicultural population. Therefore, this study, which is to our knowledge the first effort to explain health determinants by focusing on socio-environmental and individual factors, contributes to the scientific evidence for the health of AMFs population.

Our findings on the relationships between social support and well-being among AMFs stress that social support plays a key role in improving well-being. Adolescence is a period of life when social networks steadily expand through relationships with friends, but AMFs in South Korea were reported to be passive and had difficulty in making friends (14). The stronger the support system, the more it acts as a buffer, as positive emotions, self-worth, and well-being guard against psychological stress (23). In terms of family support, immigrant parents lack Korean proficiency and it may be difficult to acquire sufficient information for raising their children, leading to alienation from the family and breakdown of interpersonal relationships (33). As children grow, they may have needs to be addressed and met through communication with parents, such as career decisions and academic problems. Therefore, support should be provided by linking AMFs with community resources such as college students' mentoring, community service centers, and counseling services. Teachers' support is a key resource for adolescents' social and emotional well-being (34), and is strongly related to youths' life satisfaction (35) and well-being (23). Hence, systematic support of school staff is needed to improve the physical and psychological well-being of AMFs, and school nurses may lead school staff regarding health-related education and problem behaviors among AMFs. The governmental support system should be also established to ensure that family, school, and community provide sufficient social support through a one-stop system.

SOC (e.g., emotional connection with neighbors and feeling of belonging to the community) is a crucial development task for adolescents in order to form psychosocial bonds with the external world and develop positive feelings concerning the community (36). SOC is known to protect psychological well-being (37), and decreases depression, improves self-worth, and facilitates emotional attachment to others (38). Immigrants with stronger SOC belongingness showed higher emotional well-being (39), highlighting the importance of programs that promote links or involvement with the native population to encourage integration into society, which will enhance well-being among AMFs. In South Korea, most of community policies for multicultural families are administered separately from general native Korean population and consequently, AMFs may build limited small networks. Therefore, it will be important to apply strategies building inclusion and diversity to community programs for AMFs in connection with the native adolescent population.

This study shows that environmental (family and school) factors in the contexts of SDH, such as economic status, explain the consistencies in the previous studies on the relationship between AMFs' well-being (40). Multicultural families themselves are not the problem, but rather socioeconomic issues have a larger effect on AMFs' well-being (14), indicating a need to focus on the socioeconomic vulnerabilities of multicultural families. Given consideration of the difficulty to access health support among AMFs due to the barrier, it is crucial to provide community-level interventions in a culturally appropriate manner corresponding the needs of AMFs and their families. The current study also showed that AMFs attending general schools had higher well-being than those attending multicultural alternative schools. This is because most of the AMFs registered in general school are second-generation, while multicultural alternative schools had a high proportion of first-generation AMFs. The first-generation AMFs face a very different challenge from those of the second-generation AMFs (14), such as having to overcome various environmental difficulties, like language barriers, adaptation to a newly formed family, and adjustment to Korean culture. For the first-generation AMFs, it is necessary to establish a system of cultural adaptation to promote well-being, such as an intensive program providing support in areas such as language adaptation, education, and health care.

Acculturative stress is an important concept for immigrants and represents the stress experienced in transitioning from the native to a new culture (41). In the current study, acculturative stress did not significantly or directly affect AMFs' well-being, but the finding that the lower the social support for AMFs, the higher their level of acculturative stress, should not be overlooked. AMFs are likely to face prejudice and discrimination, which are still prevalent in Korean society but an earlier study of Joo et al. (42) showed that the perceived support of Chinese Koreans from family and meaningful others decreased the perception of acculturative stress. Therefore, an approach that improves social support from peer, teachers, or family for AMFs will be important in that mental health problems such as depression and anxiety can be triggered when acculturative stress is high (43, 44). However, as acculturative stress had a mediating role in the relationship between social support and health outcomes (42), a future study is needed to confirm the mediating effect of acculturative stress on the relationship between social support and well-being in AMFs.

There are several limitations to this study. We combined the observed variables of health behavior into exogenous variables after removing some items for SEM by factor loading. Further studies need to reconfirm the structural model by using proven and internally consistent health behavior measures. In addition, our data were collected via a convenience sampling from eight big cities in South Korea and showed that the participants presented different levels of well-being depending on residential area and type of school. Additional studies performing a large population-based multilevel analysis with AMFs after dividing them by region, school type, and immigrant generation is suggested.

## Conclusions

This study revealed that social and community network and environmental factors beyond individual factors were identified as important determinants affecting AMFs' well-being. In order to improve AMFs' well-being, it is necessary to establish integrated management linking family, school, and community. We also encourage AMFs to participate in various activities to enhance their interactions with native youth. Furthermore, the results of this study are expected to contribute to the introduction of multicultural support and policy programs that could eliminate health inequalities and gaps that AMFs face in the South Korean society.

## Data Availability Statement

The original contributions presented in the study are included in the article/supplementary material, further inquiries can be directed to the corresponding author/s.

## Ethics Statement

The studies involving human participants were reviewed and approved by Yonsei University Health System Institutional Review Board. Written informed consent to participate in this study was provided by the participants' legal guardian/next of kin.

## Author Contributions

JS and HL: design and writing manuscript. JS: preparing the questionnaire, data collection, and analyses. All authors: critical revision and approval of the final draft.

## Conflict of Interest

The authors declare that the research was conducted in the absence of any commercial or financial relationships that could be construed as a potential conflict of interest.

## Abbreviations

AMF: adolescents in multicultural families
SDH: social determinants of health
SOC: sense of community.
